# In Vitro Evaluation of Calcium Phosphate Bone Cement Composite Hydrogel Beads of Cross-Linked Gelatin-Alginate with Gentamicin-Impregnated Porous Scaffold

**DOI:** 10.3390/ph14101000

**Published:** 2021-09-29

**Authors:** Shih-Ming Liu, Wen-Cheng Chen, Chia-Ling Ko, Hsu-Ting Chang, Ya-Shun Chen, Ssu-Meng Haung, Kai-Chi Chang, Jian-Chih Chen

**Affiliations:** 1 Advanced Medical Devices and Composites Laboratory, Department of Fiber and Composite Materials, Feng Chia University, Taichung 407, Taiwan; 0203home@gmail.com (S.-M.L.); rayko1024.rb@gmail.com (C.-L.K.); s8401027@gmail.com (H.-T.C.); ya0610ya@gmail.com (Y.-S.C.); dream161619192020@gmail.com (S.-M.H.); ketty60221@gmail.com (K.-C.C.); 2 Department of Fragrance and Cosmetic Science, College of Pharmacy, Kaohsiung Medical University, Kaohsiung 807, Taiwan; 3 Dental Medical Devices and Materials Research Center, College of Dental Medicine, Kaohsiung Medical University, Kaohsiung 807, Taiwan; 4 Department of Orthopedics, College of Medicine, Kaohsiung Medical University, Kaohsiung 807, Taiwan; 5 Department of Orthopedics, Kaohsiung Medical University Hospital, Kaohsiung 807, Taiwan

**Keywords:** hydrogel, calcium phosphate bone cement, drug release, biocompatibility, antibacterial activity, in vitro

## Abstract

Calcium phosphate bone cement (CPC) is in the form of a paste, and its special advantage is that it can repair small and complex bone defects. In the case of open wounds, tissue debridement is necessary before tissue repair and the subsequent control of wound infection; therefore, CPC composite hydrogel beads containing antibiotics provide an excellent option to fill bone defects and deliver antibiotics locally for a long period. In this study, CPC was composited with the millimeter-sized spherical beads of cross-linked gelatin–alginate hydrogels at the different ratios of 0 (control), 12.5, 25, and 50 vol.%. The hydrogel was impregnated with gentamicin and characterized before compositing with CPC. The physicochemical properties, gentamicin release, antibacterial activity, biocompatibility, and mineralization of the CPC/hydrogel composites were characterized. The compressive strength of the CPC/hydrogel composites gradually decreased as the hydrogel content increased, and the compressive strength of composites containing gentamicin had the largest decrease. The working time and setting time of each group can be adjusted to 8 and 16 min, respectively, using a hardening solution to make the composite suitable for clinical use. The release of gentamicin before the hydrogel beads was composited with CPC varied greatly with immersion time. However, a stable controlled release effect was obtained in the CPC/gentamicin-impregnated hydrogel composite. The 50 vol.% hydrogel/CPC composite had the best antibacterial effect and no cytotoxicity but had reduced cell mineralization. Therefore, the optimal hydrogel beads content can be 25 vol.% to obtain a CPC/gentamicin-impregnated hydrogel composite with adequate strength, antibacterial activity, and bio-reactivity. This CPC/hydrogel containing gentamicin is expected to be used in clinical surgery in the future to accelerate bone regeneration and prevent prosthesis infection after surgery.

## 1. Introduction

Today, due to improvements in the healthcare system and advances in materials, people’s life expectancy has increased, which in turn has increased the number of patients requiring orthopedic implant surgery [1]. This has also led to an increase in the number of infections associated with orthopedic surgery, although the infection rate has declined. Implants and bone substitute materials after infection often lead to the formation of microbial biofilms, which can lead to serious complications, usually requiring debridement or complete removal of the prosthesis for a second operation. Prevention of infection is usually done through prophylactic treatment with antibiotics, however, the blood flow around the implant is usually poor, and intravenous or oral antibiotics have little success compared to in situ antibiotic therapy [2]. Polymerized polymethyl methacrylate (PMMA) is usually mixed with liquid methyl methacrylate, and then polymerized within 5 to 10 min to form an adhesive material, and can incorporate antibiotics against the target bacterial pathogen [3]. However, the antibiotic used in PMMA must be provided in powder form and stabilize the heat generated during the polymerization reaction. With 3 to 10 mm spheres, gentamicin-impregnated hydroxyapatite ceramic beads act as an osteoconductive matrix to simulate bone grafts, and unlike PMMA beads, they do not have to be removed when used to fill the space [4]. At present, there are few studies discussing the drug release of biodegradable hydrogel beads with antibiotics in combination with calcium phosphate cement (CPC) on the scale of a few millimeters.).

CPC is made by pre-mixing two or more calcium phosphate compounds with a hardening solution to make a paste, mud, or slurry that is convenient for clinical injection [5,6]. The phase of CPC after hardening is very similar to that of natural cortical bone; therefore, CPC has good biocompatibility, osteoconductivity, negligible shrinkage, non-exothermic self-setting, and injectability. CPC is suitable for filling complex bone defects in plastic surgery, dentistry, and craniofacial surgery [7,8,9]. The insufficient strength of conventional CPC can now be solved through particle surface modification or vacuum sintering, which can accelerate the hardening reaction of CPC to obtain excellent mechanical properties, injectability, and resistance to disintegration when used for blood abundance repair [10]. However, CPC with high initial strength lacks a porous structure, which prolongs its degradation time and makes the in-growth of blood vessels, osteoclasts, and osteoprogenitor cells difficult [11,12]. In addition, the ideal bone filling material promotes the activity of osteoblasts in the early stage of healing while inhibiting the growth of bacteria to prevent infection [13]. 

The most effective strategy to improve the shortcomings of the pore shortage of CPC is to add degradable hydrogels, such as alginate [14], gelatin [15], alginate/chitosan [16], hyaluronic acid [17], and silk fibroin [18]. Studies have pointed out that the addition of hydrogel particles can act as a carrier during the mixing and injection process, which means that the hydrogel protects the carrying factor from the impact of CPC hardening for a certain time [16]. In addition, the hydrogel can form pores in situ after degradation, and these pores can lead to the in-growth of blood vessels, promote the transport of progenitor cells, and accelerate tissue healing [15].

Gelatin, a structural protein composed of chains of amino acid, mainly glycine, proline, and hydroxyproline arranged in a triple helix, is very suitable for cell attachment, cell growth, and the maintenance of cell physiological functions. The degradation products can be absorbed and metabolized by the body without causing adverse cell and tissue reactions [19,20]. Gelatin can stimulate cell proliferation and promote osteoblast differentiation [21]. An in vivo study found that CPC/gelatin composite is partially biodegradable and therefore plays a role in bone replacement [22]. Alginate is composed of two uronic acids, namely, β-d-mannuronic acid and α-l-guluronic acid, which can easily undergo ion exchange reactions with divalent cations, such as Ca^2+^, Sr^2+^, and Ba^2+^, to form a chelate compound structure [23]. This structure has good biocompatibility, biodegradability, and non-toxicity and is widely used in bone grafts and drug carriers [23,24,25]. In addition, studies have composited antibiotic-impregnated alginate with CPC, and the results show that alginate can delay the release of antibiotics and stimulate the proliferation of bone cells [26]. Uncrosslinked hydrogels have problems such as high viscosity, low mechanical strength, and rapid degradation. However, the cross-linked hydrogel does not adhere, and it is difficult to fit the repair site.

Based on the CPC setting reaction at low temperatures which enable the incorporation of heat liable drugs, proteins, and hydrogels. Several studies have been performed to determine the drug loading capacity of CPCs [27]. Therefore, the release rate depends on many factors, including the hydrophobicity or hydrophilicity of the drug in the carrier, the shape, size, and morphology of the carrier, and the interaction between the drug and the polymer and copolymers or mixtures of polymers with different structures. This alternative option provides the choice of drug release during treatment. Combining CPC and antibiotic-loaded hydrogel may be an excellent way to solve these shortcomings. Our preliminary studies on gentamicin-impregnated hydrogels after cross-linking show that the hydrogels have good antibacterial activity, but their degradation resistance and biocompatibility are greatly reduced by gentamicin impregnation. The hydrogels can absorb a large amount of blood before it degrades and simultaneously releases antibiotics to prevent infection. 

In this study, gelatin/alginate hydrogel beads were used as a gentamicin carrier and composited with CPC with high mechanical properties to prepare a multifunctional composite bone cement that can be used as a bone tissue repair filler with good mechanical strength, injectability, and biocompatibility. Additionally, the antibacterial effect of gentamicin released by the composite was evaluated when the hydrogel beads were degraded. The CPC/hydrogel composite is expected to promote blood vessel growth outside the cell activity during hydrogel degradation and accelerate the repair of bone defects to increase its application value in clinical surgery in the future.

## 2. Results and Discussion

### 2.1. Characterization of CPC/Hydrogel Composites

#### 2.1.1. Hydrogel Structures, Compressive Strength, and Fracture Surface of CPC/Hydrogel Composites

**Figure 1:**
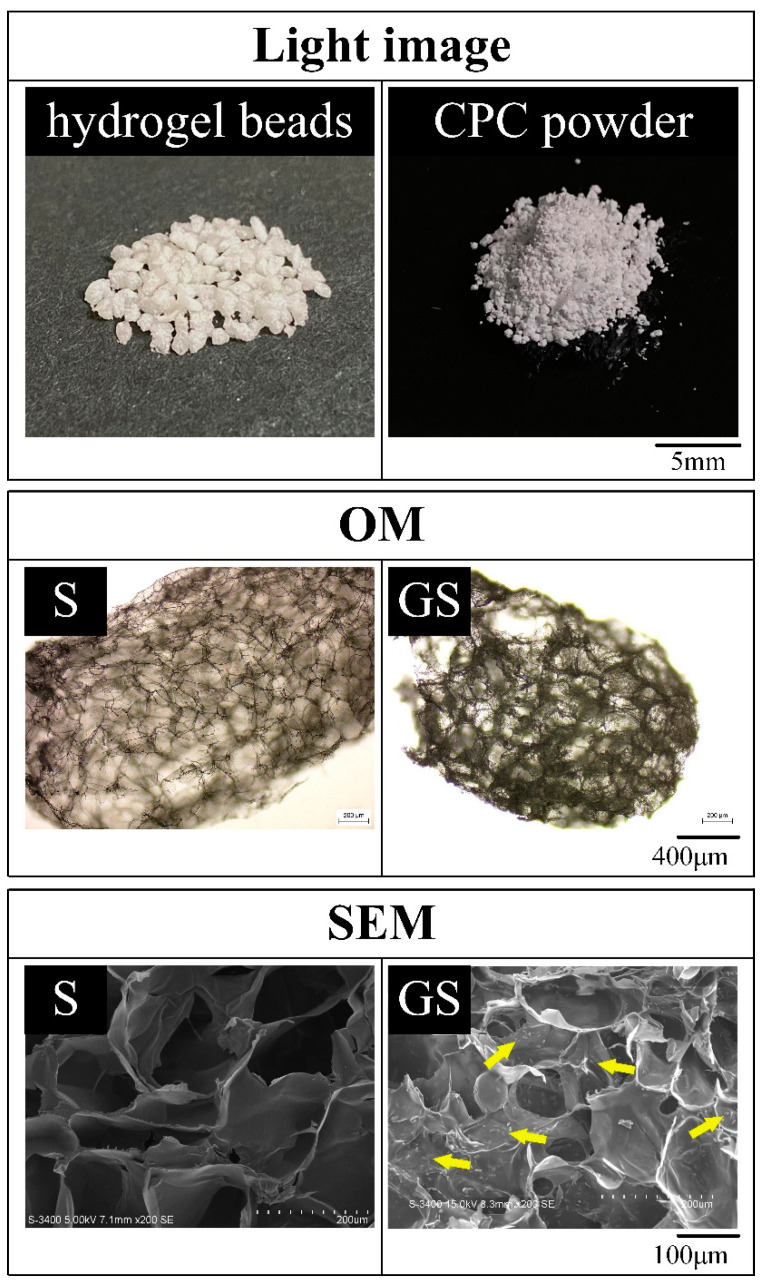
Appearance and microscopic images of hydrogel spherical beads before and after gentamicin impregnation (S: hydrogel-only, GS: gentamicin-impregnated hydrogel; yellow arrow shows the gentamicin dispersion; OM: optical images produced in the microscope; SEM: images produced by scanning electron microscope).

The appearance and microscopic images of the hydrogel beads before (S) and after immersion in gentamicin (GS) are shown in Figure 1. The dried hydrogel has an obvious porous structure with a pore size of about 200 μm. No remarkable difference was observed in the pore size of the hydrogel before and after gentamicin immersion. Gentamicin adheres to the GS pore wall, which proves that the hydrophilic hydrogel beads can successfully carry the hydrophobic drug, gentamicin.

**Figure 2:**
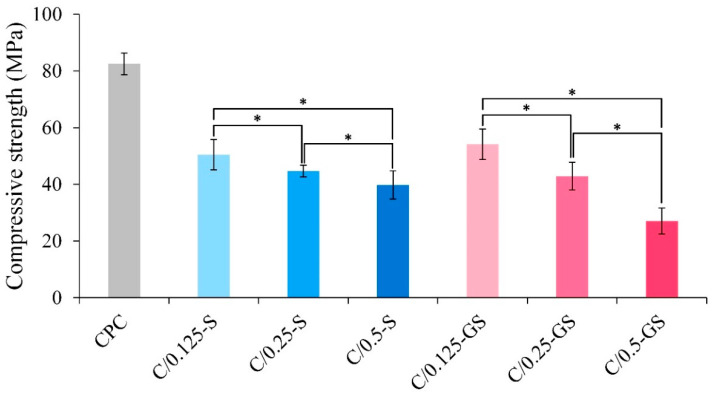
Compressive strengths of CPC/hydrogel composites after immersion in Tris-buffer solution for 1 day (*n* = 10; * indicates significant difference at *p* < 0.05).

The compressive strengths of the CPC/hydrogel composites are shown in Figure 2. The composite strength of CPC after soaking in Tris-buffer solution for 1 day is 80 MPa, whereas those of the CPC/hydrogel composites remarkably decreased, especially for the composites with high hydrogel content. The relatively high compressive strength of C/0.125-S and C/0.125-GS composites reached 50 MPa, which is still remarkably greater than that of human trabecular bone (0.1–16 MPa) [28]. According to statistical results, the compressive strength of the composites is indeed related to hydrogel content. When a large amount of hydrogel is added to the composite, uneven distribution of load and force transmission is likely to occur, resulting in stress shielding in the rigid composite matrix. The compressive strength of the C/0.5-GS group is reduced to an average value lower than that of the cortical bone (30 MPa) [10,11,12]. Therefore, the composite is not suitable for load-bearing bone repairs.

**Figure 3:**
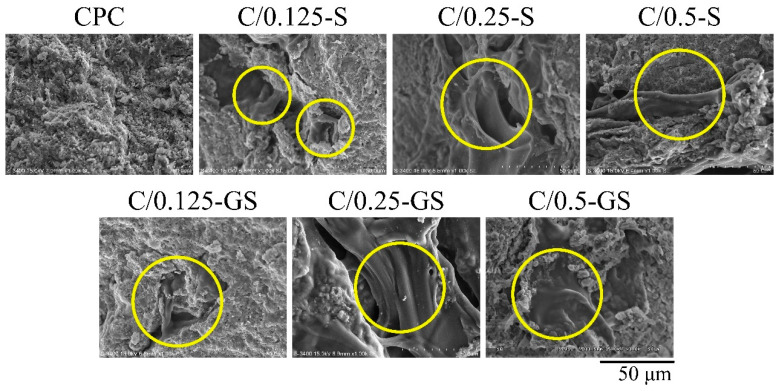
SEM fracture analysis of CPC/hydrogel composites immersed in Tris-buffer solution for 1 day. Images inside the yellow ring show that the composites contain a hydrogel.

The fracture shows that the hydrogel content group has a clear structure (Figure 3), which is consistent with the reason for the decrease in strength. Each group had a hydrogel bead squeezing effect and a coral reef-like structure, which was typical hydroxyapatite (HA) formation after CPC hardening. This morphology means that the presence of hydrogel in the CPC/hydrogel composites will not remarkably inhibit the phase transition during CPC hardening.

#### 2.1.2. Attenuated Total Reflection–Fourier Transform Infrared (ATR-FTIR) Spectroscopy and X-ray Diffraction (XRD) Patterns

**Figure 4:**
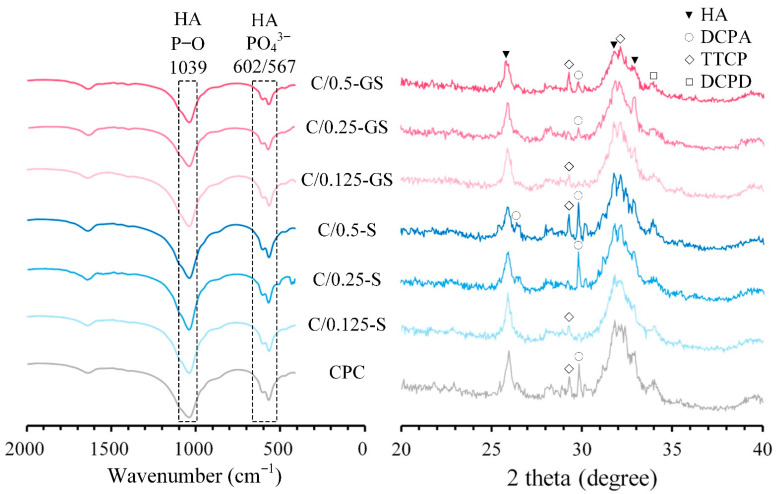
FTIR (**left**) and XRD (**right**) results of CPC/hydrogel composites after immersion in Tris-buffer solution for 1 day.

The FTIR spectra and XRD patterns of the composites are shown in Figure 4. The absorption from the ν_4_ PO_4_^3−^ bands of HA are found at 567 and 602 cm^−1^ [29]. The absorption of P–O and P=O stretching bands from the phosphate (PO_4_^3−^) of HA is characterized at 1039 cm^−1^. The FTIR spectra of the CPC/hydrogel composites and CPC-only are similar, which indicates that the hydrogel in the CPC rigid matrix does not affect the functional groups of the CPC after the hardening reaction.

When each composite was immersed in Tris-buffer solution for 1 day, the XRD patterns were compared using the files in the JCPDS database (HA, JCPD #09-0432; DCPA, JCPD #09-0080; DCPD, JCPD #09-0077; TTCP, JCPD #25-1137). The diffraction peaks of HA located at 2*θ* = 25.90°, 31.76°, 32.87° are correlated with the (002), (211), (112), and (300) crystal planes [26]. These peaks exist in the XRD patterns of the CPC/hydrogel composites and CPC-only. This result also confirms that the hydrogel in the CPC composite does not affect HA formation. In comparison, the HA diffraction peaks of the composite with gentamicin-impregnated hydrogel broadened with the increase in gentamicin. This result means that the crystallinity of HA has a downward trend with the increase in gentamicin. Thus, we speculated that gentamicin has a higher impact on the strength of the composite than hydrogel content, and the presence of alginate can prolong the release of gentamicin from CPC [26].

#### 2.1.3. Working/Setting Time, Injectability, and Dispersibility

The working and setting time of all groups were adjusted to 8 and 16 min, respectively, and the results are shown in Table 1. When a larger proportion of hydrogel beads is added to the CPC/hydrogel composite, a larger volume of hardening solution must also be added to make the working/setting time consistent with the CPC-only group. Based on the expectations of consulting clinicians and the literature [26,30,31], the ideal working time of the composite should be controlled within 4–10 min, and the setting time should be in the range of 10–20 min. The results show that the developed CPC/hydrogel composite reached the expected operation time.

**Figure 5:**
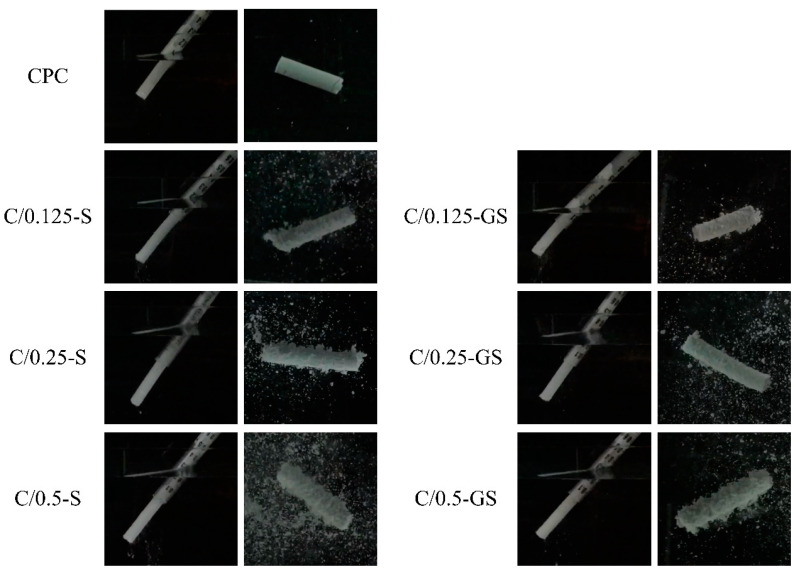
Injectability (**left images**) and anti-disintegration (**right images**) of different CPC/hydrogel composites in ddH_2_O.

The results of the injection and disintegration resistance are shown in Figure 5. CPC has good resistance to disintegration. Although the injectability of the CPC/hydrogel composites is superior to that of CPC-only because of the lubricating effect of the wetting hydrogel, its disintegration resistance slightly decreased. The ability to resist disintegration became worse as the hydrogel content increased because the presence of the hydrogel facilitates disintegration after swelling.

#### 2.1.4. Gentamicin Release from CPC/Hydrogel

**Figure 6:**
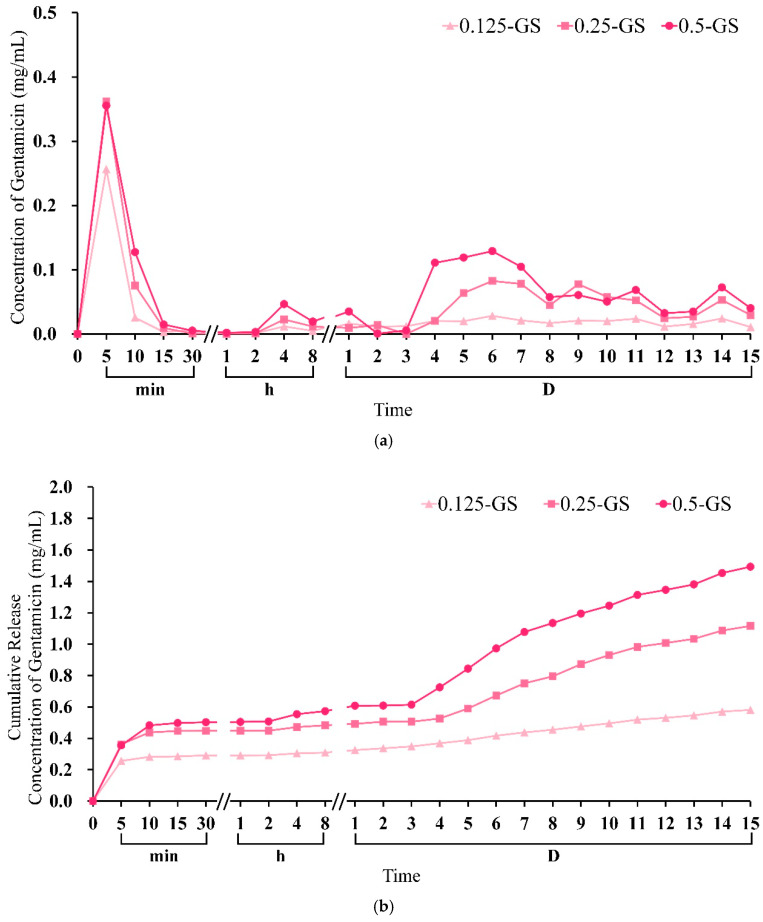

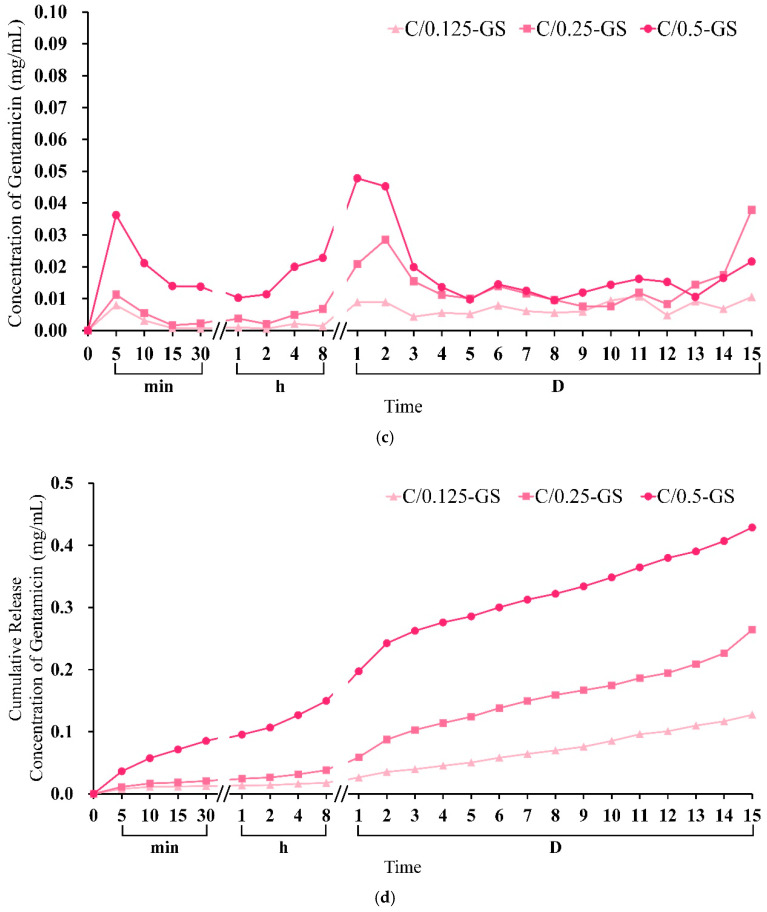
(**a**) Timely release and (**b**) cumulative release of gentamicin from different volumes of hydrogel beads at different time points; (**c**) timely release and (**d**) cumulative release of gentamicin from CPC/hydrogel with gentamicin at different time points (*n* = 3).

The timely and cumulative release results at different times before and after the gentamicin-impregnated hydrogel was composited with CPC are shown in Figure 6. The initial burst release was observed in hydrogel-only groups. Figure 6a,b show that the GS group released a large amount of gentamicin because of the swelling and degradation within 10 min and 3–11 days after soaking, respectively. This result indicates that the hydrogel beads cannot release antibiotic drugs slowly and stably. However, the gentamicin released by the CPC/hydrogel composites remarkably decreased by 10 times within 10 min, and the sustained release effect of gentamicin in the hydrogel beads was achieved after subsequent soaking for 15 days (Figure 6a,c). In addition, the C/0.125-GS and C/0.25-GS composites showed a sustained release of a small amount of gentamicin within 8 h (Figure 6d), they showed an increased release rate in 1–2 days, and finally exhibited a stable release rate. Few studies of the in vitro effects of gentamicin on osteogenic activity have been reported. The literature showed that gentamicin (0.1 mg/mL) exposure for 4 days or more has toxic effects on osteoblast-like cells [32,33]. The release of gentamicin in this study showed that only the C/0.5-GS composite had a sustained release of gentamicin exceeding 0.01 mg/mL for 4 days. Gentamicin is a typical aminoglycoside antibiotic with low molecular weight and weak binding to plasma proteins. Free access to the glomerular filtrate is the main route of excretion. Gentamicin binds to specific ribosomal proteins, resulting in the production of non-functional complexes, leading to the misreading of mRNA. After binding, gentamicin enters the cell through endocytosis, and the endocytic vesicles fuse with the phagosome [34]. Therefore, repeated administration can lead to kidney accumulation and nephrotoxicity. Although the reference range in plasma leads to a peak toxicity of gentamicin greater than 12 µg/mL, gentamicin is rapidly excreted through glomerular filtration, resulting in a plasma half-life of 2 h in patients with normal renal function, and a half-life of 100 h in the renal cortex [35]. It is recommended to administer gentamicin in-situ, allowing targeted antibiotics to be delivered locally in a high concentration. Takechi et al. disclosed the incorporation of flomoxef sodium into the apatite cement combined with chitosan [36]. Their results showed that the initial 24 h release was rapid, and the subsequent 72 h release was less pronounced, with a total of 49% released in the highest loaded samples. In addition, they also showed that adding chitosan to bone cement can reduce the initial release and increase the release of antibiotics. 

### 2.2. Antibacterial Activity, Biocompatibility, and Mineralization of CPC/Hydrogel

#### 2.2.1. Antibacterial Ability of CPC/Hydrogel with Gentamicin

**Figure 7:**
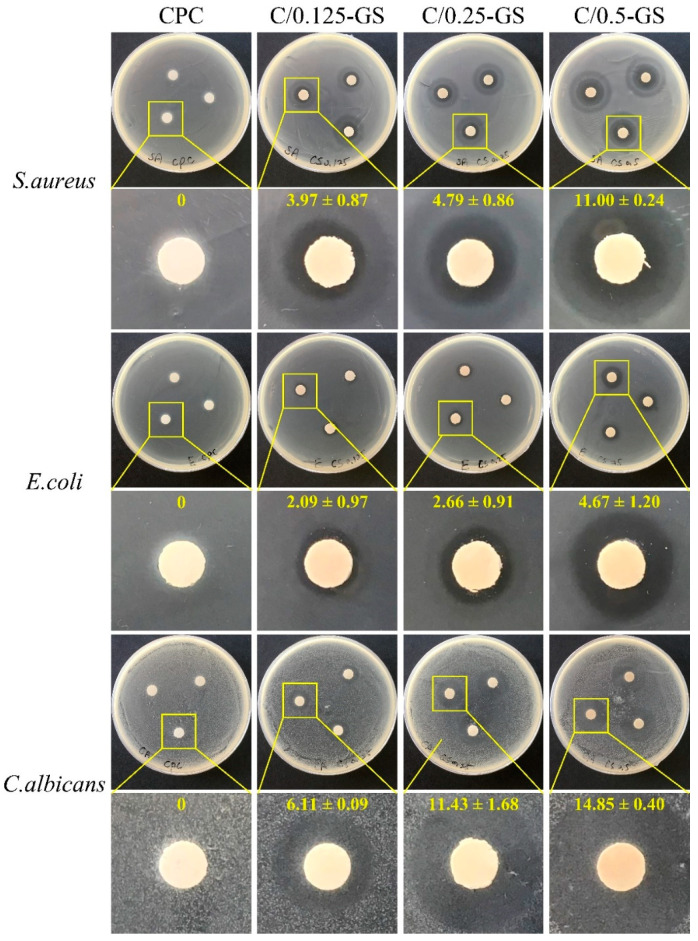
Analysis of three different bacterial inhibitory zones of CPC/hydrogels with gentamicin after 1 day of incubation (value unit: mm).

The qualitative results of the antibacterial activity of the CPC/hydrogel composites show that the antibacterial effect is better when more GS is added (Figure 7). The C/GS composite has obvious antibacterial effects on *S. aureus* and *E. coli*. For *C. albicans*, although the C/GS composite still shows a substantial inhibition zone, a fog appears on the agar plate. This fog is the residual hyphae of *C. albicans* when it undergoes apoptosis. 

**Figure 8:**
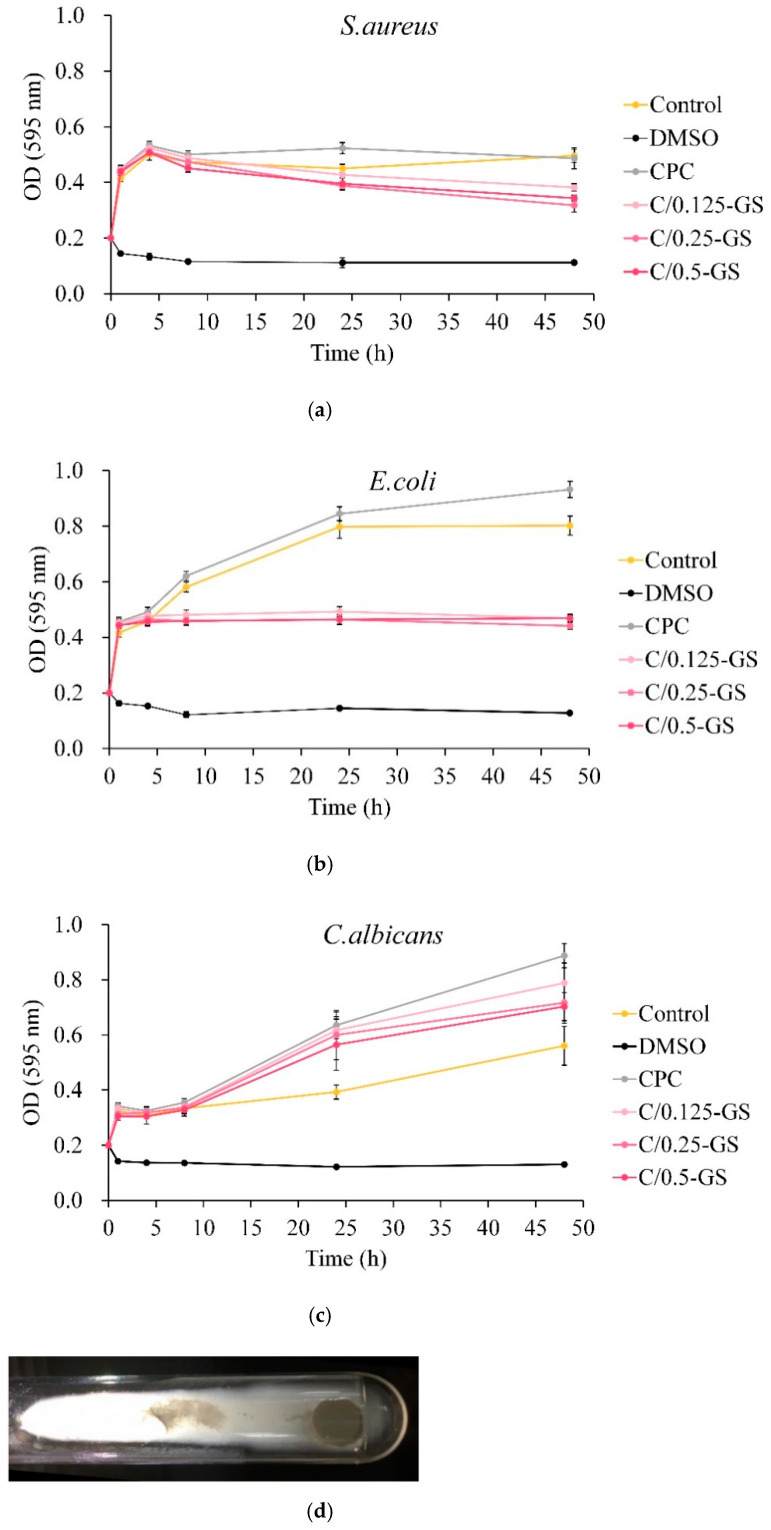
Quantitative analysis of bacterial activity inhibition by gentamicin release from CPC/hydrogel composites against (**a**) *S. aureus*, (**b**) *E. coli*, and (**c**) *C. albicans* (*n* = 3); (**d**) optical image of *C. albicans* mycelium after culture, which shows that hyphae affect the OD value reading.

The quantitative results of antibacterial activity are shown in Figure 8. Figure 8a,b display that each group has a good antibacterial effect on *E. coli* and *S. aureus* but has no obvious bactericidal effect. Figure 8c shows the antibacterial effect of C/GS on *C. albicans*. The absorbance did not flatten or decrease but increased considerably because the hyphae of *C. albicans* will inevitably be inhaled when the bacterial suspension is aspirated. This occurrence may have interfered with the recorded value. The optical image of *C. albicans* hyphae after C/GS culture shows a large number of hyphae (Figure 8d), which caused the recorded data to be higher than the actual value.

#### 2.2.2. Cytotoxicity of CPC/Hydrogel Composites

**Figure 9:**
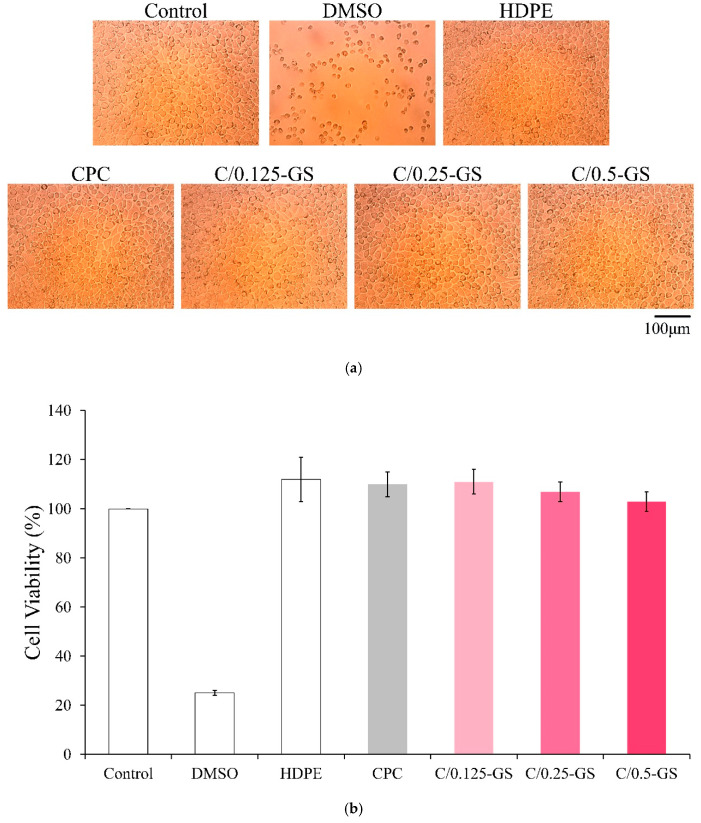
(**a**) Qualitative and (**b**) quantitative (*n* = 5) analyses of the cytotoxicity of different CPC/hydrogels with gentamicin on L929 cells.

The cytotoxicity results of C/GS extracts on cultured cells are shown in Figure 9. The amount of GS added to the composites does not affect cell viability. Thus, C/GS can promote cell growth without causing any cytotoxicity.

#### 2.2.3. D1 Cell Attachment, Proliferation, and Semi-Quantitative Mineralization

**Figure 10:**
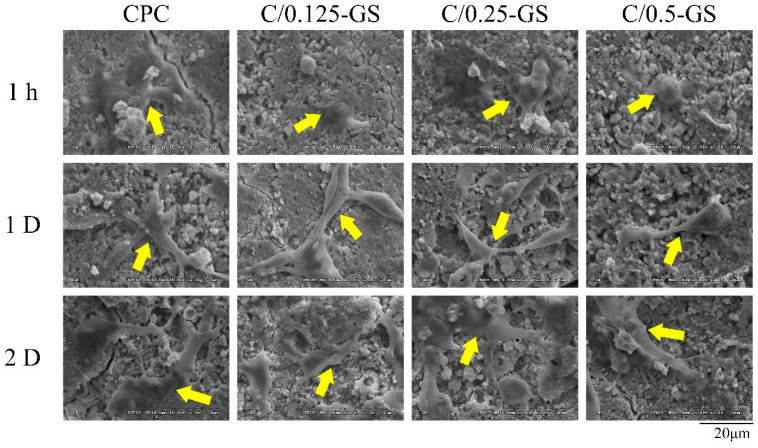
SEM images of cell attachment on CPC/hydrogel composites in contact with D1 cells for 1 h, 1 day, and 2 days.

The adhesion of osteoprogenitor D1 cells on the surface of the C/GS composites in cell contact culture is shown in Figure 10. After the cells were cultured on the sample surface for 1 h, D1 cells began to adhere to the CPC-only and C/0.25-GS sample surfaces. However, the cells on C/0.125-GS and C/0.5-GS remained spherical, which means that the D1 cell attachment in these samples was not as good as that on CPC-only and C/0.25-GS. After 1 day of culture, the cells were almost flat on the sample surface, and the pseudopodia stretched well. These changes indicate that adding GS to CPC does not affect cell attachment and proliferation. The expansion of the cytoplasm within the porous composite structure of the 3-D network after the hydrogel is degraded is important for subsequent cell proliferation [26,33,37].

**Figure 11:**
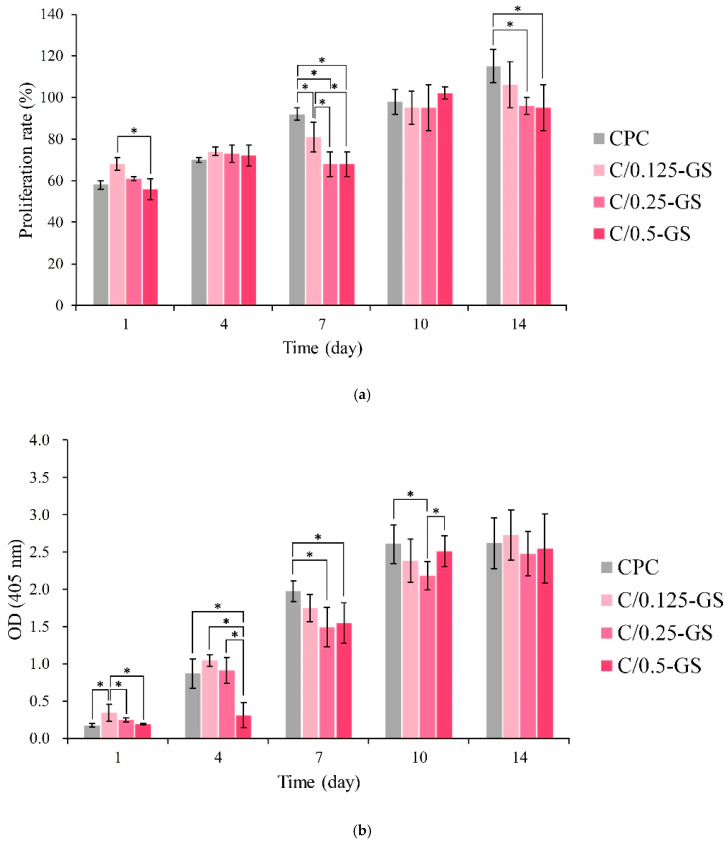
(**a**) D1 cell proliferation on CPC/hydrogel composites in different cultured time, (**b**) OD_405_ of total cell ALP activity (*n* = 3, * p < 0.05).

Figure 11a shows the quantitative results of the long-term proliferation of D1 cells in the C/GS group. The results show that the cells will proliferate, and that cell viability will also increase with the extension of culture time. When the cells were cultured for 1 day, the cell viability of the C/0.125-GS composite was the highest. On the 4th day, the cell survival rate of each group was about 60%. The cell viability of CPC did not increase considerably on the 7th day, which indicates that D1 progenitor cells began to mineralize through D1 cell differentiation. Gentamicin inhibited cell proliferation in the early stage, which resulted in relatively low cell viability. ALP could be detected in all groups on the 1st day after the D1 cells were contacted with CPC and C/GS composites (Figure 11b). Except for the C/0.5-GS group, the amount of ALP in each group increased on the 4th day. The release of gentamicin can inhibit the ALP expression of D1 cells; thus, the high release of gentamicin can inhibit the cell viability and differentiation of D1 cells in the early stage. Alginate, gelatin collagen, and hyaluronic acid contain hydrophilic groups, such as –OH, –COOH, –NH_2_, and –CONH–, and can be similar to the extracellular matrix; thus, they can promote the binding of major integrin and cell adhesion [26,38]. Calcium phosphates also can enhance cell attachment and osteogenic differentiation; thus, only hydrophobic gentamicin can negatively affect osteogenic function in vitro. In the literature, gentamicin (12.5–800 μg/mL) was remarkably reduced by one-third to one-half compared with the control [32]. Therefore, under the positive influence of hydrogel and CPC combined with the negative influence of gentamicin, the present study achieved compromising results in the osteogenic function of CPC/hydrogel with gentamicin.

## 3. Materials and Methods

### 3.1. Raw Materials

The raw materials used in this study include tetracalcium phosphate (Ca_4_P_2_O_9_, TTCP; Realbone Technology Co., Ltd., Tainan, Taiwan), surface-modified dicalcium phosphate anhydrous (sm-DCPA; Realbone Technology Co., Ltd., Tainan, Taiwan), gelatin (80–100 Blooms (USP-NF, BP, Ph. Eur.) pure, pharma grade; PANREAC, EU), alginic acid sodium salt from brown algae (low viscosity; Sigma-Aldrich^®^, St. Louis, MO, USA), *N*-(3-dimethylaminopropyl)-*N*’-ethylcarbodiimide hydrochloride (EDAC; molecular weight, 191.70 g/mole; Sigma-Aldrich^®^, St. Louis, MO, USA), calcium chloride (CaCl_2_; Shimakyu Chemical Co., Osaka, Japan), gentamicin (Siu Guan Chemical Industrial Co., Ltd., Chiayi, Taiwan), and sucrose (Katayama Chemical Co., Ltd., Osaka, Japan).

### 3.2. In Situ Preparation of CPC Composite with Hydrogel Beads

#### 3.2.1. CPC Bone Cement Preparation

The original preparation processes of TTCP and sm-DCPA were referenced from the literature [10]. CPC powder was prepared by mixing 16.6 g TTCP with an average particle size of 12.6 μm and 12.4 g of sm-DCPA with an average particle size of 2.0 μm. The concentration of the hardening solution (NaH_2_PO_4_) was adjusted to 0.67 M, and the final pH value was 6.02. The CPC powder was mixed with the hardening solution with different powder-to-liquid (P/L) ratios to obtain a cement slurry. The P/L ratio of the CPC/hydrogel composites depends on the required working time, which was set to 8 min, and the setting time was set to 16 min (Table 1).

#### 3.2.2. Hydrogel Bead Preparation

Gelatin and sodium alginate were at a ratio of 4:1 (*g*/*g*), stirred in double-distilled water (ddH_2_O) at a ratio of 10 g/mL, and heated to 50 °C for 30 min to prepare a colloidal suspension. The suspension was then mixed with sucrose with a controlled particle distribution of 90–212 μm at a particle-to-colloid ratio of 4 g/mL. The principle of the mixing ratio is that more than 70% of the interconnected pores in the gel beads are formed after the particles are leached. Automatic input was used to inject the colloidal solution into 100 mL of the solution where the EDAC/CaCl_2_ cross-linking agent was mixed in a constant weight percent ratio of 1:0.5 at a certain pushing speed of 1 mL/min. After the beads were cross-linked and hardened into semi-finished hydrogel beads for 24 h, the semi-finished hydrogel beads were further immersed in 1 wt% EDAC solution to completely leach the sucrose. Then, the porous hydrogel beads were washed with ddH_2_O for 3 h and freeze-dried (FDU-830, EYELA, Tokyo, Japan) for 24 h to obtain spherical hydrogel bead carriers named as the S group. A 0.1 g of the hydrogel bead carrier was soaked in 5 mL of 40 mg/mL gentamicin solution for 30 min, frozen for 3 h, and then freeze-dried for 24 h. The prepared spherical hydrogel carrier containing gentamicin was noted as the GS group.

### 3.3. Characterization of CPC/Hydrogel Bead Composites

#### 3.3.1. Compressive Strength and Fracture Surface Observation

The paste was filled into a stainless-steel mold to form a 12 mm-high and 6 mm-diameter cylindrical specimens for compressive strength testing. In addition, 3 mm-thick and 6 mm-diameter discs were prepared for subsequent tests. Samples were prepared by mixing different CPC/hydrogel composite groups with the hardening solution for 1 min and then filled into the mold within 10 min. The CPC/hydrogel composite in the mold was held at 0.7 MPa for 2 min and then demolded. The test method was conducted following ASTM F451-16. The demolded sample was immersed in an artificial body fluid (Tris/HCl-buffer solution) with a ratio of 1 g/10 mL) at 37 °C for 24 h before testing. A desktop universal mechanical tester (LS 500, Lloyd Instruments, Tokyo, Japan) was used to measure the compression of the wet sample at a crosshead speed of 1.0 mm/min, and the maximum strength value was recorded. Afterward, the tested sample was immersed in 99.8% alcohol to terminate the reaction and dried for the microstructure analysis of the fracture surface by scanning electron microscopy (SEM; S-3000N, Hitachi, Tokyo, Japan). Then, the dispersion of the hydrogel beads inside the composites was observed.

#### 3.3.2. ATR-FTIR Spectroscopy and XRD Analyses

The impregnated composite sample was ground and mixed with KBr at a ratio of 1:100 to form a translucent disc. Then, the sample was placed in a FTIR instrument (Nicolet 6700, Thermo Fisher Scientific, Waltham, MA, USA) to observe whether new functional groups were generated.

An X-ray diffractometer (XRD-6000, Shimadzu, Kyoto, Japan) was used to obtain the diffraction patterns of the CPC/hydrogel composites after immersion. XRD was run at 30 kV and 20 mA using Ni-filtered Cu target Kα diffraction at the scanning speed of 2°/min. The XRD patterns with scanning angles between 20° and 40° were collected. The phases were identified by comparing the diffraction patterns with standard files in Joint Committee on Powder Diffraction Standards.

#### 3.3.3. Working/Setting Time, Injectability, and Dispersibility

Working time was measured in the paste without adhesion when the CPC/hydrogel mixed solution started to harden. Setting time was measured according to ISO 9917-1 at 37 °C and relative humidity of 60–70%. A Vicat needle (400 g, 1 mm diameter) was used to press the surface vertically. Setting time is reached during solidification if no obvious indentation appears on the surface and the indentation depth does not exceed 1 mm.

The CPC/hydrogel composite was mixed for 2 min, placed in a 1 mL needle-free syringe for 2 min, and pushed into a transparent container filled with ddH_2_O. Then, the appearance of the composite in water was observed.

#### 3.3.4. Antibiotic Release Testing by Ultraviolet/Visible (UV-Vis) Spectrometry

The relationship between light absorption value and analyte concentration can be expressed by Beer–Lambert’s law as follows:

(1) $$A=\log\left(\frac{I_{0}}{I}\right)=\varepsilon bc$$

where *A* is the absorbance value, *I*_0_ is the incident light intensity, *I* is the transmitted light intensity, *ε* is the molar absorption coefficient, *b* is the optical path length (cm), and *c* is the molar concentration of the sample to be tested. UV–vis spectroscopy (UV1800, Shimadzu, Kyoto, Japan) was used to detect the gentamicin-impregnated hydrogel bead and its composite CPC/hydrogel. The optical density (OD) value of gentamicin at the wavelength of 190–220 nm was proportional to the release amount of CPC/hydrogel composites. A gentamicin calibration curve was constructed, and the measured value of the test product was converted to the release amount of gentamicin at each time point. The samples of different groups were immersed in 4 mL of ddH_2_O at 37 °C. The drug release at different test times was detected by UV–Vis spectrometry.

### 3.4. Antibacterial Activity, Biocompatibility, and Mineralization of CPC/Hydrogel with Gentamicin after Sterilization

#### 3.4.1. Antibacterial Ability of CPC/Hydrogel with Gentamicin

Staphylococcus aureus, Escherichia coli, and Candida albicans were selected to test the antibacterial potential of the CPC/hydrogel composites by agar diffusion test. Antibacterial activity was assessed in qualitative discs (3 mm thick, 6 mm diameter) using the well diffusion technique. The medium used was tryptic soy broth agar (Neogen, Lansing, MI, USA). Tryptic soy broth was also used as the medium for the quantitative analysis of the influence of gentamicin-impregnated hydrogels on drug release and antibacterial activity. The strains were cultured at 37 °C without CO_2_ to avoid bacterial overgrowth.

The antibacterial qualitative test was performed as follows. The CPC/hydrogel samples were sterilized with 25 kGy gamma-ray radiation. Then, the disc was prepared, pasted on a bacteria-coated agar culture plate, and incubated at 37 °C for 24 h. The size of inhibition was measured. For antibacterial quantification, the disc was immersed in 1 mL of bacterial suspension and incubated at 37 °C for 24 h. The absorbance of the bacterial suspension at OD_595_ was measured using an enzyme-linked immunosorbent assay (ELISA) reader (EZ Read 400, Biochrom, Cambridge, UK). The absorbance at OD_595_ is directly proportional to bacterial activity.

#### 3.4.2. Cytotoxicity of CPC/Hydrogel Composites

Cytotoxicity test was carried out under ISO 10993-5:2009 for the biological evaluation of the CPC/hydrogel with gentamicin. The cell culture media were all from Gibco (Thermo Fisher Scientific Inc., Waltham, MA, USA). The fibroblast cell line, L929, was used and cultured in modified Eagle’s medium containing house serum in an incubator containing 5% CO_2_ at 37 °C. The medium was changed every 2 days under normal cell culture. The L929 cells in the control group were cultured in a normal cell culture medium. The positive control was 15% dimethyl sulfoxide filtered through a 0.22 μm filter membrane. The negative control was high-density polyethylene extract with a weight-to-medium volume ratio of 1 g/10 mL.

Each group was quantitatively tested (*n* = 6), and the sterilized composite discs of CPC/hydrogel with gentamicin were placed in a medium at 37 °C at a ratio of 1 g/10 mL for 24 h to prepare an extract. The cells were transferred into a 96-well microliter plate at a concentration of 1 × 10^4^ cells/well and cultured overnight in an incubator. Then, the original medium was aspirated, and the hydrogel extracts (100 μL/well) were used to cultivate the L929 cells for 24 h. The medium was then aspirated, and a general cell culture medium (100 μL/well) was then added, mixed with a cell proliferation assay kit (XTT; 50 μL/well; Biological Industries, Kibbutz Beit Haemek, Israel), and cultured for 4 h. Subsequently, OD_490_ was measured because the OD_490_ measured by XTT is directly proportional to cell viability. Cell morphology was also observed using an optical microscope (CK, Olympus, Tokyo, Japan).

#### 3.4.3. D1 Cell Attachment, Proliferation, and Semi-Quantitative Mineralization

Short-term cell attachment, proliferation, and alkaline phosphatase (ALP) activity were quantified on D1 cells, a cell line of mouse precursor osteoblasts. The cell culture used was Dulbecco’s modified Eagle medium containing 10% fetal bovine serum. The disc sample was cultured in contact with D1 cells with a cell density of 1 × 10^4^ cells/well. The test disc was placed in a 48-well plate and incubated for 1 h, 1 day, and 2 days. Then, the sample was washed with phosphate buffer saline (PBS), fixed with a mixture of 2.5% glutaraldehyde and paraformaldehyde, and dehydrated in a gradient alcohol sequence. The specimen was plated with metal, and cell adhesion morphology was observed by SEM.

In addition, the disc samples were cultured in contact with D1 cells at a density of 1 × 10^5^ cells/well in a 48-well plate. The cultivation time was 1, 4, 7, 10, and 14 days. Then, the cell culture medium was mixed with Alamar Blue proliferation assay kit (Bio-Rad, Hercules, CA, USA) to wash and culture the sample for 4 h. OD_570_ and OD_595_ were measured with an ELISA reader.

### 3.5. Statistical Analysis

ANOVA and two-sample *T* test in IBM SPSS Statistics version 20 (IBM, New York, NY, USA) were used to discuss and analyze differences in compressive strength, cell proliferation, and ALP secretion capacity. A *p*-value less than 0.05 indicates a significant difference.

## 4. Conclusions

Antibiotic-impregnated hydrogel beads combined with CPC is an effective strategy to control the sustained and stable release of gentamicin. It has no cytotoxicity and does not reduce osteogenic mineralization. In this study, the C/0.125GS and C/0.25-GS composites showed a compressive strength of 40–50 MPa, which did not affect its injection performance but deteriorated its anti-disintegration ability. The working time and setting time of the composites can reach 8 and 16 min, respectively, which are in line with clinical expectations, by adjusting the content of the hardening solution in the CPC/hydrogel composite. All C/GS composites can effectively control the release of gentamicin. Thus, the C/GS composites have a linearizing effect on the release curve, which is beneficial to bone restorations that require long-term treatment. However, the C/GS composites have reduced strength and the HA phase of the product formed by CPC is also slightly reduced because of the properties of the gentamicin-impregnated hydrogel. All C/GS composites, even C/0.5-GS, have obvious antibacterial effects, good cell adhesion properties, and good biocompatibility. The C/0.5-GS composite inhibited the ALP production of D1 cells on the 4th day, but this phenomenon did not appear in the other groups. This result indicates that excessive GS in CPC would inhibit the differentiation ability of ALP. Therefore, C/0.25-GS can be selected as the optimal composite with high compression resistance, stable and sustained drug release ability, good antibacterial activity, and good biocompatibility. This research can increase the clinical applicability of CPC/hydrogel composites and the development of future multifunctional applications, as CPC/gentamicin-impregnated hydrogel beads can release antibiotics for a long time and fill the repair site to promote bone formation.

## Figures and Tables

**Table 1:** Designated names of CPC composite hydrogels with different volume ratios, measured work/setting time, and relative powder-to-liquid ratio.

Named Groups	Working Time/Setting Time (Mean ± Standard Deviation; *n* = 10; Unit: min)	Adjusted Powder-to-Liquid Ratio (g/mL)
CPC	8.49 ± 0.11/16.79 ± 0.14	0.8/0.280
C/0.125-S	8.43 ± 0.07/16.70 ± 0.13	0.8/0.285
C/0.125-GS	8.47 ± 0.09/16.82 ± 0.09	
C/0.25-S	8.42 ± 0.06/16.72 ± 0.13	0.8/0.290
C/0.25-GS	8.43 ± 0.07/16.80 ± 0.12	
C/0.5-S	8.39 ± 0.05/16.74 ± 0.16	0.8/0.300
C/0.5-GS	8.44 ± 0.07/16.80 ± 0.10	

## Data Availability

Data is contained within the article.
